# Essential Oil Stabilisation by Response Surface Methodology (RSM): Nanoemulsion Formulation, Physicochemical, Microbiological, and Sensory Investigations

**DOI:** 10.3390/molecules27217330

**Published:** 2022-10-28

**Authors:** Mariem BenJemaa, Fatma Zohra Rahali, Hanen Falleh, Raja Beji-Serairi, Wided Megdiche-Ksouri, Ibtissem Hamrouni, Majdi Hammami, Ralph Nehme, Renato B. Pereira, Sonia Andrés, Said Bouhallab, David M. Pereira, Fabrizio Ceciliani, Latifa Abdennebi-Najar, Riadh Ksouri

**Affiliations:** 1 Laboratory of Aromatic and Medicinal Plants, Biotechnology Center of Borj-Cédria BP 901, Hammam-Lif 2050, Tunisia; 2 INRAE, Institut Agro, STLO, F-35042 Rennes, France; 3 Quality and Health Department, IDELE Institute, 149 Rue de Bercy, CEDEX 12, 75595 Paris, France; 4 REQUIMTE/LAQV, Laboratory of Pharmacognosy, Department of Chemistry, Faculty of Pharmacy, University of Porto, R Jorge Viterbo Ferreira 228, 4050-313 Porto, Portugal; 5 Instituto de Ganadería de Montaña, CSIC-Universidad de León, Finca Marzanas s/n, 24346 Grulleros, Spain; 6 Department of Veterinary Medicine, Università degli Studi di Milano, 20122 Milan, Italy; 7 Centre de Recherche Saint-Antoine (CRSA), Sorbonne University, INSERM UMR_S_938, 75020 Paris, France

**Keywords:** essential oil, nanoemulsion, RSM experimental design, physical characterisation, biological activities, sensory analysis

## Abstract

This manuscript aimed to optimise the encapsulation of *Thymus capitatus* essential oil into nanoemulsion. Response Surface Methodology results were best fitted into polynomial models with regression coefficient values of more than 0.95. The optimal nanoemulsion showed nanometer-sized droplets (380 nm), a polydispersity index less than 0.5, and a suitable Zeta potential (−10.3 mV). Stability results showed that nanoemulsions stored at 4 °C were stable with the lowest *d*_3,2_, PolyDispersity Index (PDI), and pH (day 11). Significant ameliorations in the capacity to neutralise DPPH radical after the encapsulation of the antimicrobial efficacy of thyme essential oil were recorded. *S. typhimurium* growth inhibition generated by nanoencapsulated thyme essential oil was 17 times higher than by bulk essential oil. The sensory analysis highlighted that the encapsulation of thyme essential oil improved enriched milk’s sensory appreciation. Indeed, 20% of the total population attributed a score of 4 and 5 on the scale used for milk enriched with nanoemulsion. In comparison, only 11% attributed the same score to milk enriched with bulk essential oil. The novel nanometric delivery system presents significant interest for agroalimentary industries.

## 1. Introduction

The emergence of pathogenic organisms resistant to antibiotics is one of the greatest concerns facing the health system today [1]. Multidrug-resistant (MDR) bacterium infections are becoming more widespread and pose a severe threat to public health. Originally, these species were only found in hospitals, but now they can be found elsewhere [2]. The causes primarily responsible for the spread of antibiotic-resistant species include globalization, excessive use of antibiotics in animal husbandry and aquaculture, the use of numerous broad-spectrum drugs, and a lack of adequate antimicrobial stewardship [3]. Antibiotic-resistant diseases are becoming more common, which means there are fewer antimicrobial medicines available to treat the illnesses caused by these bacteria. According to Vivas, Barbosa, Dolabela and Jain [1], if no new drugs are created or found by 2050, there will be no effective antibiotics available to treat illnesses [4].

This necessitates the quest for alternative antibiotic-resistant disease control approaches, and numerous research groups around the world are actively exploring such answers.

Scientists studying the properties of essential oils (EOs) have made interesting discoveries on the potential of these secondary metabolites to control microbial contamination. Indeed, the in vitro biological efficiencies of some EOs (mainly antioxidant, antimicrobial, and anti-inflammatory activities) have strengthened their candidacy as a viable weapon against antibiotic-resistant bacteria, particularly in pathologies where the aetiological agents have biofilm structures [5]. Besides their obvious benefits for pharmacology, these EOs are an excellent choice for the food industry as natural antimicrobial agents capable of protecting easily alterable matrices, such as dairy products, from microbial spoilage [6]. EOs, which represent less than 5% of the vegetable dry matter, are volatile, liquid, and colourless, with low water solubility but high solubility in alcohol, organic solvents, and fixed oils [6]. However, these molecules present some physical properties limiting their value in phytosanitary and veterinary products or in the food industry, including their sensitivity to light, enzymes, temperature, oxygen, and pH fluctuation, in addition to their low stability [7]. Moreover, the selection of potent EOs that does not alter food aroma, taste, and flavour is one of the main limitations of their use as food preservatives [7]. As a result, innovative industries have begun to adopt novel procedures based on the encapsulation of the molecules of interest. This approach aims to protect EOs from the environment, lowering their toxicity and masking their strong flavour and taste. In this context, different techniques are proposed, mainly the encapsulation of EOs into nanoemulsion-based delivery systems [8]. EO hydrophobicity can be reduced when trapped in nanoemulsions, rendering their utilisation easier and more feasible. Moreover, the encapsulation of EOs into a nanoemulsion-based delivery system appears to improve their biological efficacy in more accurate food models [9]. It is also important to highlight that nanoemulsions are thought to have superior antibacterial activity than conventional emulsions with much larger droplet sizes due to their nano-sized droplets, which enhance the active surface area [10].

Forming a nanoemulsion-based delivery system is a complex phenomenon depending on chemical and physical parameters [8]. The selection of ingredients (type and concentration) raises a significant challenge to obtaining the best physical stability and the highest biological efficiency of the produced nanoemulsions. Indeed, the selection of the suitable mixture of the dispersed phase (pure EOs, or EOs mixed with fixed oils) and the continuous phase composition (type and proportion of the emulsifier) represents a critical point in controlling the suitability of the formulated nanoemulsions to deliver their enclosed bioactive molecules [11]. Pongsumpun, et al. [12] reported that choosing to formulate nanoemulsions with either low-energy or high-energy methods depends on the formulated nanoemulsion applications. Consequently, the formulation step must be carefully optimised to obtain a stable nanoemulsion with efficient biological activities by considering any eventual influencing parameters’ variation effects and interactions. However, considering these numerous variables via the traditional experimental approach is costly and time-consuming [12]. Numerical approaches, such as the response surface methodology (RSM), might be employed instead to reduce the challenges of traditional experimentation [13]. RSM is a statistical and mathematical technique employing a second-degree polynomial model to investigate the links between one or more response variables and numerous independent factors using either univariate or multivariate models [14]. In this context, Pierlot, et al. [15] have proposed the Hadamard matrix for the optimisation screening of a significant number of factors *X*_i_, (*i* ≥ 4) that can potentially influence the response *Y*.

This manuscript’s objective was to optimise the encapsulation of *Thymus capitatus* EO (TEO) into a nanoemulsion-based delivery system (TNE) and evaluate its in vitro properties and efficacy against pathogenic microorganisms. According to an RSM plan, the formulation was subjected to a physicochemical characterisation. The encapsulation effect on the biological activity was assessed by comparing the antiradical and antimicrobial potential of bulk and nanoencapsulated EO. Finally, a sensorial hedonic analysis was performed to identify whether subjects appreciated the encapsulation effect on enriched milk.

## 2. Results and Discussion

GC/MS chemical analyses of the *T. capitatus* essential oil allowed the identification of 15 components, representing 99.4% of the total GC/MS peak areas. The major fraction of Thyme EO was constituted by carvacrol which was distinguished as the major compound representing more than 70% of the total EO components, distantly followed by γ-terpinene and *p*-cymene at 7.6% and 7%, respectively (Table S1).

### 2.1. TEO Nanoemulsion Formulation

#### 2.1.1. Model Fitting and Statistical Analysis

According to the Hadamard combination design principle, a response surface method was created using NemrodW software DesignExpert 8.05b to optimise the encapsulation of TEO into a nanoemulsion-based delivery system. The used experiment design included 4 independent variables and 11 runs. The experimental and predicted droplet size diameters (*d*_3,2_) of the proposed nanoemulsions are listed in Table 1.

The experimental particle size of formulation 5 was 463 nm, which was the smallest of all 11 experiments, as shown in Table 1. The particle size of formulation 6 was 1012 nm, the highest in all formulations. Conjointly, a good symmetry can be observed when comparing the experimental results with the predicted ones for the 11 runs.

NemrodW software was also used to calculate the model coefficients, estimate the variance analysis of nanoemulsion droplet sizes, and perform mathematical and graphical analyses of the gathered results. The obtained data from the RSM were fitted to the second-order polynomial equation. The regression coefficients of different independent variables and the corresponding *p*-values are illustrated in Table S2 in the Supplemental Materials.

In fact, the statistical significance of the regression model was checked by F-test using NemrodW software (Table S2). The statistical obtained results of the validated mathematical model exhibited that only independent variables presented significant effects, since their Ficher F-test of regression coefficients were superior to the tabulated values, and the *p*-value was smaller than 0.001. A deeper analysis highlights that factors *β*_0_, *β*_1_, and *β*_2_ were considered significant. In contrast, the factors *β*_3_ and *β*_4_ are moderately significant, confirming that the variation factors and their interactions significantly influence the targeted response. However, the effects of the interaction between the model variables were not significant since their Ficher F-test of regression coefficients were inferior to the tabulated values, and the *p*-value was superior to 0.001 (data not shown). Accordingly, the regression equation proposed by NemrodW should be composed of only the independent tested variables.

The data detailed in this table exhibited that for the studied response (*d*_3,2_), all independent variables have significant quadratic effects (*p* < 0.01). A deeper analysis highlights that factors *β*_0_, *β*_1_, and *β*_2_ were considered significant. In contrast, the factors *β*_3_ and *β*_4_ are moderately significant, confirming that the variation factors and their interactions significantly influence the targeted response. However, the effects of the interaction between the model variables were not significant since their Ficher F-test of regression coefficients were lower than the tabulated value, and the *p* value was higher.

The validity of the elaborated model was confirmed through lack of fit testing (ANOVA) using the Fisher test, as described in Table S3 in the Supplemental Materials. The F-test value of regression coefficients is superior to the tabulated (F_regression_ ≥ F_tabulated_ (6, 4, 0.01)). The corresponding *p*-value (<0.0001) indicates that independent factors significantly affect droplet diameter size. Furthermore, results from the same table indicated that the lack-of-fit is not statistically significant (F_lack-of-fit_ < F_tabulated_ (4, 2, 0.01)), which means that the model is valid. Hence, the model is valid and convenient for predicting the mean droplet diameter of TNE under any combination of values of the variables.

**Figure 1:**
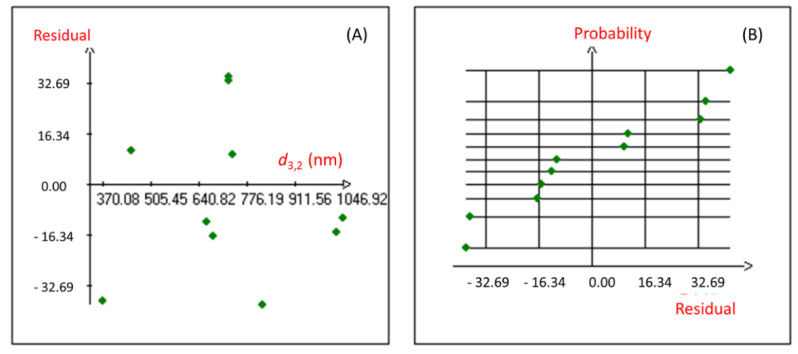
Graphical study of the residuals of the nanoemulsion droplet size diameter. (**A**): Graphic study of residuals; (**B**): Normal Plot of residuals.

Moreover, the coefficient of multiple determination (r^2^) of 0.981 was obtained for the studied response, revealing a good correlation between the response and the independent variables. The proposed Hadamard model was also confirmed using the statistical validation of the fitted model. Indeed, the graphical study of the *Y* response residuals (Figure 1) showed that the distribution of the points from the experimental and calculated values of the studied model is random, and no particular structure emerges from the representation of the residuals.

Therefore, the regression equation proposed by NemrodW (Equation (1)) can be used to describe the encapsulation of TEO into a nanoemulsion-based delivery system, the actual relationships between every tested factor (percentage of emulsifier in the continuous phase; stirring time; sonication time; dispersed phase percentage) and the observed response (droplet size diameter, *d*_3,2_), and to ascertain the optimum conditions.

*Y*(*d*_3.2_) = 724.10 − 129 × *X*_1_ + 183.27 × *X*_2_ + 25.30 × *X*_3_ + 15.60 × *X*_4_ (1)

with *X*_1_: Percentage of the continuous phase; *X*_2_: Stirring time; *X*_3_: Sonication Time; and *X*_4_: Percentage of the dispersed phase.

**Figure 2:**
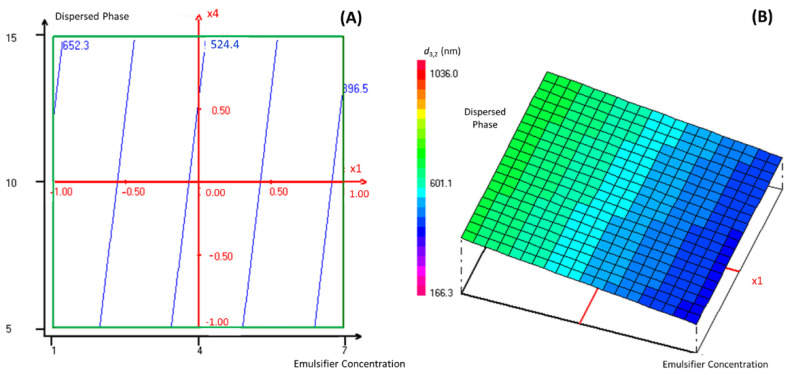
The two- dimensional (**A**) and three-dimensional (**B**) representations of the responses of the effects between the emulsifier percentage in the continuous phase and the dispersed phase percentage on the droplet size diameter of formulated nanoemulsions when the stirring and the sonication time were fixed at a level of 0.

As this manuscript’s objective is to encapsulate TEO into a nanoemulsion-based delivery system with the lowest possible droplet size diameter, the regression coefficients of the predicted second-order polynomial model were used to predict the most significant parameters that influence the studied response. Indeed, according to Table S2, the emulsifier percentage in the continuous phase and the dispersed phase percentage were chosen for the response surface analysis of TEO nanoencapsulation, as they presented the lowest regression coefficients. In this context, Figure 2 presents graphically the two- and three-dimensional representations of the responses of the significant interaction effects between the emulsifier percentage in the continuous phase and the dispersed phase percentage on the droplet size diameter of the formulated nanoemulsions when the stirring and the sonication time were fixed at level 0, which corresponds to 3 and 15 min, respectively.

Accordingly, the droplet size diameter of the formulated nanoemulsion decreased significantly (reaching the value of 396.5 nm) with the increase in the emulsifier percentage in the continuous phase and the decrease in the dispersed phase percentage. It was established in previous work [16] that the emulsifier concentration influences the formation of nano-scale range droplets significantly. Indeed, the emulsion formation depends on the sufficient interfacial tension decrease between the two immiscible phases. Using the appropriate emulsifier at a convenient concentration can ensure the necessary decrease in the interfacial tension [17]. Accordingly, Tween emulsifiers, as hydrophobic and hydrophilic structures, link the gap between the dispersed phase (TEO) and the continuous phase by decreasing the interfacial tension at the oil-water interface. Thus, Pongsumpun, Tang and Wongvanich [12] declared that the increase in Tween concentration leads to the formation of smaller particle sizes, and that the optimal droplet size could be obtained when the emulsifier is used at its critical micellar concentration, where the interfacial tension between the two immiscible phases reaches a stable state.

It is important to highlight that despite their significant effect on emulsion droplet diameter, the use of either of the tested high-energy methods was not the main factor in the optimal TEO encapsulation into nanoemulsion. The low-energy emulsification process was based on the self-emulsification method for this case study. This method involves the slow addition of the dispersed phase into the aqueous phase and the reliance on the rapid diffusion of the emulsifier molecules from the oil phase to the continuous phase, leading to small-emulsion-droplet formation [18].

In this context, Pongsumpun, Tang and Wongvanich [12] considered that the sonication bath container wall acts as an ultrasonic wave barrier causing wave mitigation before reaching the sample. Concerning EO encapsulation using high-speed homogenisation, Cui, et al. [19] were concerned about the rate of emulsion coalescence caused mainly by high temperatures induced during the high-speed homogenisation.

#### 2.1.2. TEO Encapsulation into Nanoemulsion-Based Delivery System Using the Desirability Approach

To optimise the encapsulation of TEO into the optimal stable nanoemulsion with the lowest droplet size diameter, the four significant factors in the regression equation were first-order partial derivatives. The desirability analysis was a bilateral Derringer transformation, and the target diameter was set at 270 nm. The optimal coding values of the factors for solving the equations were *X*_1_ = 1; *X*_2_ = −1; *X*_3_ = −1, and *X*_4_ = −1. Accordingly, the optimum preparation conditions were as follows: the percentage of the emulsifier in the continuous phase: 7%; the stirring time: 0 min; the sonication time: 0 min; and the percentage of the dispersed phase: 5%. The predicted response (*Y*) was equal to 270 nm. The experimental verification was carried out under optimised conditions. The experiment was repeated three times, and the average droplet size diameter of the obtained nanoemulsions was 260.8 ± 1.52 nm. The measured droplet size diameter was close to the predicted value, indicating that the proposed model was reliable with accurate prediction results.

### 2.2. TEO Nanoemulsion Physic-Chemical Characterisation

Once the formulation of the TEO encapsulation into the nanoemulsion-based delivery system was statistically validated, the optimised *T. capitatus* nanoemulsion (TNE) was characterised by various physicochemical parameters such as droplet size average diameter, pH, viscosity, turbidity, Zeta potential, conductivity and refractive index, as detailed in Table 2.

Information on the average droplet diameter (*d*_3,2_) is indispensable for characterising nanoemulsions [10]. The data in Table 2 indicated a low droplet diameter (inferior to 400 nm) for the studied nanoemulsion suggesting that it has good stability. In addition to the small diameter, the optimal nanoemulsion droplet size distributions confirmed its stability since the PDI measurement was inferior to 0.5. The viscosity measurement of the optimal TNE demonstrated a very low value (0.1 mPa/s), positively confirming its stability since stable nanoemulsions are characterised by their low viscosities [10]. According to Falleh, Jemaa, Neves, Isoda, Nakajima and Ksouri [10], a lower viscosity can match the smaller average droplet size of the nanoemulsion, confirming its stability. In this respect, [20] highlighted that emulsion viscosity not only depends on the nature of emulsion components (oil, water, surfactants) and their concentrations but also on the use of physical force to obtain the nanoemulsion. Indeed, these authors demonstrated that nanoemulsions made of the same composition but through different emulsification procedures presented different viscosities. Concerning pH measurement, results demonstrated that TNE presented an acidic character with a pH value limited to 5.1.

Interestingly, our data are different from those of El Azim and Balah [21], who studied the formulation of *T. capitatus* nanoemulsion but found different pH values (pH = 7.7). The detected differences could be attributed to inorganic co-surfactant (chloroform) use in their formulation. The refractive index evaluates the light propagation through a nanoemulsion-based delivery system and its eventual transparency. The data resumed in Table 2 exhibited that TNE presented a refractive index equal to 1.35. These results agreed with TNE’s non-transparent visual aspect considering that the recorded refractive index was higher than water (1.333). The turbidity measurement of TNE indicated a turbidity of about 20.51, which was in agreement with the findings of Nirmal, et al. [22] who noted nanoemulsion’s ability to scatter light less efficiently than the larger droplet emulsion. Indeed, Falleh, Jemaa, Neves, Isoda, Nakajima and Ksouri [10] declared that nanoemulsion turbidity tends to increment its mean particle diameter. Finally, the Zeta potential is one of the most important parameters to assess nanoemulsion stability [23]. The optimal TNE presented a Zeta potential of −10.30 mV, which can be considered an excellent demonstration of the nanoemulsion’s good homogeneity and physical stability.

### 2.3. TEO Nanoemulsion Stability Assessment

**Figure 3:**
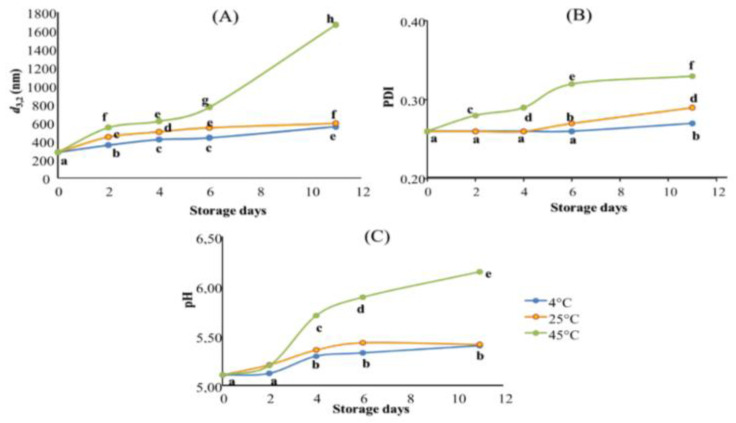
Stability assessment of TNE stored for 11 days at three different temperatures (4, 25 and 45 °C). Results are expressed in terms of (**A**): droplet size measurement (nm); (**B**): PDI values, and (**C**): pH values. Data presented as mean of three repetitions. Values followed by different letters mean statistically significant differences (*p* < 0.01).

The effect of storage conditions on the physical stability of the optimal TNE was evaluated for 11 days at three different temperatures (4, 25, and 45 °C) by monitoring their droplet size diameter (*d*_3,2_, nm), their PDI, and their pH (Figure 3).

The overlay of all stability assessment results highlighted that TNE stored at 4 °C showed the best stability during the tested storage period, followed by TNE stored at 25 °C. Finally, TNE stored at 45 °C demonstrated the worst stability (Figure 3). When it comes to their stabilities, nano-sized emulsions frequently exhibit unusual behaviour. Depending on the formulation, production techniques, and storage conditions, they can be stable for a few hours to several months after production [24]. In this context, Gupta, et al. [25] have distinguished the different physical phenomena responsible for the alteration of nanoemulsion stability, such as flocculation and coalescence (causing the droplet diameter to increase); Ostwald ripening (causing PDI increase); creaming and sedimentation (causing phase separation). These authors have also explained that the listed alteration phenomena could occur individually or could be the results of each other, depending on the nanoemulsion formulation and production process and the storage conditions. Indeed, the actual tendency to be unstable as temperature rises is an expected result. Previous scientific data stipulated that low-energy-produced nanoemulsions using non-ionic surfactants tend to be thermally sensitive [26]. Accordingly, the droplet size diameter results highlighted that TNE stored at 4 °C presented the best stability with a slight increase until the sixth day of storage (Figure 3A). Indeed, when TNE was stored at 4 °C, *d*_3,2_ values increased slowly from 279.93 nm on day 0 to 435.73 nm on day 6 and reached 556.17 nm on day 11. Despite this observed increase, TNE stored at 4 °C presented the smallest droplets during all stability assessment periods. However, the TNE stored at 45 °C showed the worst stability since their droplet size diameters increased from day 2 to reach 1666 nm on day 11 of storage. The droplet size increase in nanoemulsions during the storage period could be caused by coalescence. In the case of nanoemulsion alteration by coalescence, the droplets merge into each other to become a more significant drop, which explains the increase in *d*_3,2_ values during the storage [27]. Coalescence occurs whenever the ionic strength of the continuous phase increases, which affects the repulsive barrier between two droplets [25]. Accordingly, [8] declared that the increase in the thermal agitation of TEO droplets by the storage temperature leads to their interfacial tension decreasing, causing the droplets to flocculate and then coalesce.

Moreover, the increased storage temperature caused a significant increase in PDI values during the tested storage period. Indeed, TNE stored at 4 and 25 °C were perfectly stable for 4 days with no significant increase (*p* > 0.01) in their PDI values. However, on day 6, the PDI of TNE stored at 25 °C increased significantly (*p* < 0.01) to reach 0.27, while TNE stored at 4 °C remained stable with no significant changes in its PDI values. Only on the 11th day of storage did the PDI of TNE stored at 4 °C increase significantly, reaching 0.27 (Figure 3B). It is worthy of note that the TNE stored at 45 °C was subjected to a significant PDI increase (*p* < 0.01) from the second day of storage and continued increasing to reach the highest PDI on the 11th day of storage (PDI = 0.33). The gathered results suggested that the PDI increase in nanoemulsions during the storage period could be caused by Ostwald ripening. Indeed, Gupta, Eral, Hatton and Doyle [25] suggested that bigger droplets grow at the expense of smaller ones during the Ostwald ripening phenomenon, inducing the polydispersity of droplets to increase. In this context, Delmas, Piraux, Couffin, Texier, Vinet, Poulin, Cates and Bibette [24] highlighted that, in nanoemulsion alteration by Ostwald ripening, the mass transfer is strictly dependent on the solubility of the dispersed phase in the continuous phase, which is temperature-dependent.

Concerning the pH variation during the stability assessment period, the obtained results exhibited significant changes (*p* < 0.01) after only 4 days of storage at the three tested temperatures (Figure 3). Indeed, during the first two days of storage, the pH of the TNEs stored at 4 and 25 °C was stable with no significant changes. Initially, at the beginning of the experiments, the pH of the three TNE was 5.1. After four days, the pH of the TNEs stored at 4 and 25 °C increased to reach 5.29 and 5.36, respectively. On the contrary, pH variation during the stability assessment period was more critical when TNE was stored at 45 °C. Indeed, the pH of this TNE increased to 5.7 on day 4 and continued increasing to reach 6.15 by the end of the testing period. The gathered results suggested that TNE coalescence had led to a pH increase. With this respect, Pearson’s correlation test demonstrated a robust positive correlation between the *d*_3,2_ and pH variations (r^2^ = 0.832). Cheng, et al. [28], who studied the variation of the acidity of Isopropyl myristate *o*/*w* nanoemulsion over the same length of time, have also observed an increase in pH in the studied nanoemulsion throughout the experimentation period, which was convoluted with droplet size increase, and consequently resulted in nanoemulsion instability.

### 2.4. The Encapsulation Effect on TEO Biological Activities

It has repeatedly been proven that the nanoencapsulation of EOs can improve their biological activities, mainly the antimicrobial ones [29]. Once the optimal formulation of TEO encapsulation into a stable nanoemulsion-based delivery system was theoretically (*via* the software) and experimentally validated, the antiradical and antimicrobial activities of bulk and nanoencapsulated EOs were assessed.

#### 2.4.1. Antiradical Activity Measurements

The encapsulation effect on TEO antiradical activity was assessed using the DPPH inhibition capacity. Bulk and nanoencapsulated TEO were evaluated at the same concentration (300 µg/mL). Table 3 demonstrates that bulk and nanoencapsulated TEO presented remarkable capacities (>40%) to scavenge the DPPH radical. *T. capitatus* EO has been often linked to significant antiradical activity mainly due to its high concentration of carvacrol, exceeding 75% of the total identified molecules [9].

Considering the encapsulation effect on the antiradical capacity of TEO, the results of Table 3 demonstrated significant improvement (*p* < 0.01) of TEO’s capacity to scavenge DPPH^.^ radical after being encapsulated into a nanoemulsion-based delivery system. Indeed, the inhibition capacity was enhanced significantly (*p* < 0.01) from 40% for bulk TEO to 44% for TNE. The improvement of thyme EO activity in the NE system (4% increase) can be associated with the increased solubility of the hydrophobic EO components (carvacrol, *γ*-terpinene and *p*-cymene) due to the emulsification process [30]. In other words, activity was enhanced due to the increase in the total contact area with the phase surrounding. With this respect, some EOs are reported to act as co-surfactants in nanoemulsion systems owing to their positioning at the oil-water border, which may explain their improved antiradical activity compared to the bulk oil [30]. The gathered findings are promising for the future, especially since previous studies on the encapsulation of TEO into nanoemulsion-based delivery systems [30], while managing to produce stable nanoemulsion, found its antiradical efficiency diminished. Various reasons could explain such effects; the type and concentration of the emulsifier used and the encapsulation process (whether a chemical-only or a physicochemical process) are both responsible for the droplet size average and the biological efficiency of the produced nanoemulsion. In this study, the resort to an RSM numerical approach for TNE formulation has allowed the production of a stable nanoemulsion with enhanced antiradical activity.

#### 2.4.2. Antimicrobial Activity Measurements

The encapsulation effect on TEO antimicrobial activity was assessed using the disc diffusion method against four pathogenic bacteria and one *Candida* strain. The analysis depicted in Table 4 exhibited that bulk TEO (at the concentration of 33 mg/mL) displayed slight to moderate antimicrobial activity against the five-tested microorganisms, with inhibition zone (IZ) of 6 and 8.7 mm (against *Salmonella typhimurium* and *Enterococcus faecalis*, respectively) and 7.7 mm against *Candida albicans*. As previously discussed [30], carvacrol, the major compound of *T. capitatus* EO, is probably responsible for the observed antibacterial activity. Indeed, carvacrol (phenolic monoterpenoid) displays its antimicrobial activity through the disintegration of the microbial outer membrane, followed by the release of lipopolysaccharides and the increase in ATP permeability in the cytoplasmic membrane [8], all of which affect cell viability and lead to death.

Interestingly, in general, the antimicrobial efficacy of TEO was ameliorated after its nanoencapsulation (Table 4). The IZ generated by TNE in the inhibition test were significantly larger (*p* < 0.01) than those generated by the bulk EO (except for *P. aeruginosa*). For example, the growth inhibition of *Staphylococcus aureus* raised from 7 mm when generated by bulk TEO to 10.33 mm when generated by TNE. For better data exploitation, the percentage of the augmentation of the IZ was calculated and detailed in Table 4.

The improvement of TEO antimicrobial activity after its nanoencapsulation was quite significant and varied from 9% (*Pseudomonas aeruginosa*) to 61% in the case of *Candida albicans.* Against *Salmonella typhimurium* and *Staphylococcus aureus,* the amelioration was estimated to be 44 and 47%, while against *Enterococcus faecalis,* the IZ was augmented by around 20%.

**Figure 4:**
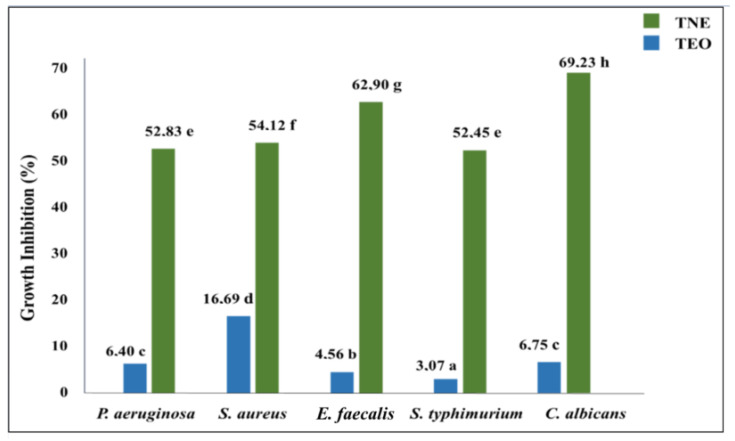
Microbial growth inhibition (expressed in %) induced by bulk (TEO) and nanoencapsulated (TNE) *T. capitatus* EO (33 mg/mL) against five microbial strains. Values followed by different letters mean statistically significant differences (*p* < 0.01).

The microplate bioassay was used to determine the encapsulation effect on the antimicrobial activity of *T. capitatus* EO against the tested food-borne pathogens (Figure 4). The obtained results indicated that bulk TEO presented moderate antimicrobial efficiency at 33 mg/mL since its growth inhibition percentage was limited to between 3 and 17% against *S. typhimurium* and *S. aureus*, respectively. The measurement of the growth inhibition percentage confirmed the antimicrobial efficacy enhancement of TEO after its nanoencapsulation, which was depicted by the inhibition zone diameter test. For instance, the *S. typhimurium* growth inhibition percentage was 17 times higher when *T. capitatus* EO was encapsulated in a nanoemulsion-based delivery system (Figure 4).

The data obtained from the antimicrobial assay confirm the remarkable interest in properly encapsulating *T. capitatus* EO into the nanoemulsion delivery system. The observed improvement in the efficacy of TEO (33 mg/mL) to inhibit microbial growth after its nanoencapsulation could be explained by the refinement of the mechanism of action of the nanoencapsulated EOs in fighting pathogenic microorganisms. Indeed, EOs exert antimicrobial activity from the cytoplasmic membrane’s inner side [8]. However, their cell penetration could be limited due to their known hydrophobicity, which can noticeably decrease their potency. In this context, some researchers consider nanoemulsion as a path for EOs to overcome their hydrophobicity limitation, allowing them to permeate the microbial cell membrane [31]. When encapsulated into nanoemulsion, EO droplets are evenly distributed in the aqueous medium, rendering them more accessible to the microbial cell wall [5,8]. Accordingly, Falleh, Jemaa, Neves, Isoda, Nakajima and Ksouri [10] considered nanoemulsions as well-established antimicrobial delivery vehicles, which can ensure the proper release of encapsulated bioactive chemicals from oil droplets leading to the improvement of the microbicidal action of the emulsified antimicrobials.

### 2.5. The Encapsulation Effect on TEO Sensorial Impact on UHT Milk Enrichment

This manuscript’s objective was to optimise the encapsulation of TEO into a nanoemulsion-based delivery system to increase its biological activity and mask its powerful sensorial impact once incorporated into the food model. Hence, the encapsulation effect on TEO sensorial impact on UHT milk enrichment was assessed using a sensory hedonic test of milk’s sensorial attributes. The obtained results were statistically treated by orange data mining software.

**Figure 5:**
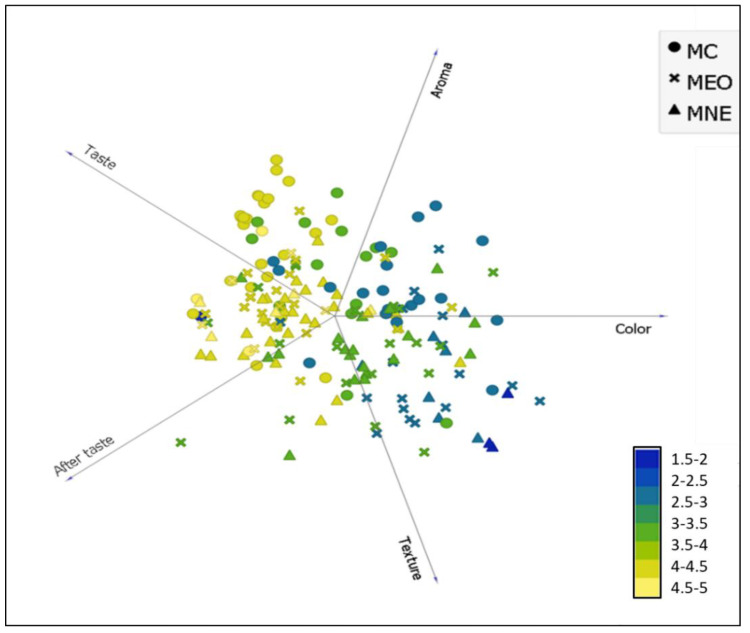
The general hedonic appreciation distribution of tested milk samples is based on their sensory attribute assessment (colour, aroma, taste, aftertaste, and texture). MC: Control milk, MEO: Milk enriched with bulk EO (1.5 mg/mL), MNE: Milk enriched with nanoemulsion (1.5 mg/mL).

The data presented in Figure 5 highlights that the general appreciation of the three tested milk samples was strongly influenced by the aroma, taste, and aftertaste of milk. However, the appreciation of the colour and texture of the milk did not significantly influence the general appreciation. Indeed, for the three milk samples, the yellow colour indicating higher general appreciation (scores > 4 on the appreciation scale used) was almost limited to flavour attributes. The blue colour indicating low milk appreciation was concentrated between the appreciation of the milk’s colour and texture. Benjemaa, et al. [32] highlighted that odour and taste are inextricably linked. The tongue’s gustatory buds identify flavour, whereas the olfactory nerves identify odours. These sensations are communicated to the brain, which integrates the information and allows you to recognise and appreciate flavours. Comparably, Tahsiri, et al. [33] stated that the barberry-enriched milk appreciation was rated according to the following order of importance: taste > aroma > colour. Therefore, these results can play a significant role in optimizing milk enrichment with TEO by evaluating the encapsulation effect on the flavour attributes.

The results in Table 5 demonstrate that the highest population (38%) scored 4 for general milk appreciation. Milk enriched with TNE received better appreciation among this population than milk enriched with bulk TEO. Indeed, 15% of the total population attributed a score of 4 on the hedonic scale used for milk enriched with nanoencapsulated TEO. In comparison, only 10% attributed a score of 4 for milk enriched with bulk TEO. The obtained results suggested that the encapsulation of TEO into a nanoemulsion-based delivery system had succeeded to mask the sensory effect of TEO once incorporated into the milk samples and consequently improved the sensory appreciation of enriched milk. Interestingly, the gathered results demonstrated that milk enriched with nanoencapsulated TEO was more appreciated by the questioned panellists than the control milk since the distribution of panellists who had attributed ≥ 3 in general appreciation scores was more significant compared to the population who had the same appreciation for the control milk (29% and 23%, respectively).

## 3. Materials and Methods

### 3.1. Plant Material, EO Extraction and GC-MS Analysis

About 3 kg of *Thymus capitatus* aerial parts, at the vegetative stage, were collected from a naturally diversified mountain in the Northeast of Tunisia (27 m altitude, 37°02′ N, 10°59′ E). All samples were shade dried for 15 days at room temperature. The dried material (200 g) was cut into small pieces and subjected to hydrodistillation using a Clevenger-type apparatus for 3 h at 80 °C. The EO was collected and stored at −20 °C in amber vials.

A gas chromatograph (HP 5890-SERIE II, Agilent, Waldbronn, Germany) coupled to a mass spectrometer (HP-MSD 5972 A, Agilent, Waldbronn, Germany) equipped with an HP INNOWAX polar column (30 m 0.25 mm, film thickness, 0.25 lm) was used to analyse the chemical composition of the *Thymus capitatus* EO. The adopted protocol was as previously described by Ben Jemaa et al. [20]. The component identification was based on comparing retention times with those of authentic standards, comparing their linear retention indices to the series of n-hydrocarbons, and by computer matching against commercial libraries (NIST 98 and ADAMS 95) and a home-made library of mass spectra built up from pure substances and components of known essential oils and the MS literature data. Peaks contributing to the total area by more than 0.01% were identified.

### 3.2. TEO Encapsulation into Nanoemulsion-Based Delivery System (TNE)

#### 3.2.1. Nanoemulsion Parameters

The encapsulation of TEO was carried out as an oil-in-water (O/W) nanoemulsion. The dispersed phase was made of a binary mixture of 70% TEO and 30% corn oil [30], while the continuous phase was made of aqueous solutions of Tween 20. Two physical forces were tested for the emulsion conception: high-speed homogenisation and ultrasonic agitation. Indeed, an UltraTurrax agitator (IKA-T25 digital, IKA^®^-Werke, Staufen, Germany) was used at 10,000 rpm, and an Ultrasonicator (Sonorex Digitec DT, BANDELIN electronic, Berlin, Germany) was used at a fixed operation frequency of 40 kHz. The specific parameters influencing the nanoemulsion stability, such as homogenisation duration and phase proportions, were elaborated using an RSM plan.

#### 3.2.2. Response Surface Methodology Experimental Design

An RSM was used to optimise the emulsification parameters of TEO. Thereby, 4 independent variables were assessed: (*X*_1_) percentage of the emulsifier in the continuous phase varied in the range of 1–7%; (*X*_2_) stirring time ranged from 0–6 min; (*X*_3_) the sonication time varied from 0–30 min; and (*X*_4_) the percentage of the dispersed phase was 5–15%. All of the listed independent variables were run at three levels for each of the individual coded values (−1, 0, and 1). The coded values (*x*_i_) of the parameters can be obtained by using Equation (2):

*x*_i_ = (*X*_i_ − *X*_0_)/Δ*X* (2)

where *X*_i_ is the actual value for the independent variable, *X*_0_ is the actual value for the independent variable at the center point, and Δ*X* is the step of change values for the independent variable. Actual levels and coded factor levels of the four independent variables for optimising the formulation of optimum TEO nanoemulsion using RSM are shown in Table 6.

A response surface plane, spherical type, 1st degree Hadamard was applied to study the effect of the independent variables (*X*_1_, *X*_2_, *X*_3_, and *X*_4_) on the response: diameter of the dispersed oil droplets (*d*_3,2_). Eleven experimental runs were required to optimise the nanoemulsion droplet size, including 3 centre points (Table 7).

A second-order polynomial equation was applied to indicate the responses that were a function of the independent variables (Equation (3)):

*Y* = b_0_ + b_1_ × *X*_1_ + b_2_ × *X*_2_ + b_3_ × *X*_3_ + b_4_ × *X*_4_ (3)

where *Y* is the response function of the dispersed oil droplet diameter (*d*_3,2_), b_0_ is a constant coefficient in Equation (1), and b_1_, b_2_ b_3_, and b_4_ are linear coefficients of *X*_1_, *X*_2_, *X*_3_ and *X*_4_ factors, respectively. Once the fitted model was statistically validated, a desirability study was conducted to theoretically provide the optimum TEO nanoencapsulation.

### 3.3. TEO Nanoemulsion Droplet Size Measurement

The droplet size of TEO nanoemulsions was measured using a particle size analyser (Zetasizer Nano ZS90, Malvern Instruments Ltd., Malvern, UK) with a helium-neon gas laser (633 nm) and a detector angle of 173°. To eliminate inter-particulate contact and multiple scattering, each sample was diluted in a 1:100 ratio with deionised water. The samples were maintained in sealed amber bottles at 25 °C, and the TEO nanoemulsions were characterised in less than 1 h. Three repetitions of the droplet size measurements were carried out.

### 3.4. TEO Nanoemulsion Physicochemical Characterisation

Once formulation was statistically validated, the optimised nanoemulsion was characterised for its physicochemical parameters. Firstly, the droplet average diameter, the polydispersity index (PDI), the Zeta potential, and the conductivity of the optimal *T. capitatus* nanoemulsion (TNE) were measured using the Zetasizer software (version 7.03). Secondly, pH measurements were determined by dipping the electrode directly into the nanoemulsion with a digital pH meter (MP220, Mettler Toledo, Greifensee, Switzerland.) at an ambient temperature of 25 °C. The refractive index evaluation was made without dilution at an ambient temperature (25 ± 2 °C) through a pocket refractometer (Atago 3810, PAL-1, Saitama, Japan). TNE turbidity was assessed at 600 nm using a UV–visible spectrophotometer (Lambda 35 UV/Vis Spectrophotometer, PerkinElmer Co, Waltham, MA, USA) after suitable dilution with pure water, and viscosity was evaluated by a rotative-viscometer (Selecta STS-2011, JP SELECTA, Barcelona, Spain). All the measurements were performed at least in triplicate at ambient temperature (25 ± 2 °C).

### 3.5. TEO Nanoemulsion Stability Assessment

The emulsion stability was investigated by storing the nanoemulsions at 4, 25, and 45 °C [8]. Measurements were taken at 0, 2, 4, 6, and 11 days of storage. The kinetic stability was determined by checking changes in droplet diameter (*d*_3,2_), PDI, and pH values, as described in the previous section, at different time intervals.

### 3.6. Encapsulation Effect on TEO Biological Activities

#### 3.6.1. Antiradical Activity

The DPPH^.^ technique was used to test the in vitro antiradical activity of bulk and nanoencapsulated TEO [30]. Aliquots of 250 µL of DPPH (0.22 mM in methanol) were added to 1 mL of each TEO sample diluted in pure methanol (300 µg/mL). The mixture was allowed to react in the dark for 30 min before being measured at 517 nm against a negative control. The results were determined as a percentage of the inhibition, which is calculated using the formula Equation (4):

IC = (DO control − DO sample/DO control) × 100(4)

where IC: inhibition capacity; DO control: absorbance of the control; and DO sample: absorbance of the examined TEO sample.

#### 3.6.2. Antimicrobial Activity

To assess the encapsulation effect on TEO antimicrobial activity, two methods were used against different pathogenic strains, namely: *Staphylococcus aureus* (ATCC 25923), *Enterococcus faecalis* (ATCC 29212), *Salmonella typhimurium* (ATCC 14028), *Pseudomonas aeruginosa* (ATCC 27853), and the fungal strain *Candida albicans* (ATCC 10231).

Qualitative evaluation of the antimicrobial activity. Each tested microorganism’s inoculum was streaked onto Mueller-Hinton agar plates for bacteria and Sabouraud agar plates for yeast using a sterile swab. After 30 min, filter discs, impregnated with 10 µL of TEO or TNE (corresponding to 33 mg/mL of 10% aqueous DMSO solution), were deposited on the inoculated agar. The plate dishes were incubated at 37 °C for 18–24 h, and the diameter of the inhibitory zones around each disc was measured after incubation.

Each experiment was repeated six times, and the average diameter of the inhibitory zone was measured in millimetres [34].

Quantitative evaluation of the antimicrobial activity. The antimicrobial activity of bulk (TEO) and nanoencapsulated (TNE) *T. capitatus* EO was expressed as the percentage of growth inhibition as described by Falleh, et al. [35]. Moreover, 25µL of either TEO or TNE (corresponding to 33 mg/mL of 10% aqueous DMSO solution) was added to a convenient broth medium in sterile 96-well plates. Subsequently, 10 µL of new microbial suspension was dropped in each well to obtain a final cell concentration of 10^6^ CFU/mL. The microbial suspension was used alone with a broth medium as a negative control. Finally, microplates were aseptically agitated and incubated for 18–24 h at 37 °C. After the incubation, microorganism growth was evaluated by reading the absorbance level at 405 nm using a microplate spectrophotometer (EZ Read 2000, Biochrom, Cambridge, UK).

The absorbance data allowed for the calculation of the percentage of growth inhibition using the formula (Equation (5)):

Growth inhibition (%) = ((A_GC_ − A_Sample_)/A_SC_) × 100 (5)

where A_GC_: absorbance of the growth control (negative control); and A_Sample_: absorbance of the samples.

### 3.7. Encapsulation Effect on TEO Sensorial Impact

UHT milk was selected as a food matrix to assess the encapsulation effect on TEO’s organoleptic properties due to its important consumption worldwide and its vulnerability toward spoilage microorganisms. Standard untreated UHT milk and UHT milk supplemented with either TEO or TNE (1.5 mg/mL) were exposed to a blind sensory evaluation to assess the encapsulation effect of TEO’s sensorial impact on UHT milk enrichment. The sensory analysis involved 76 panellists (10 men, and 66 women), aged from 20 to 40 years old (students, supervisors and staff from the Biotechnology Center of Borj-Cedria, Tunisia). The samples were stored at 15 °C and served in half-filled 20 mL pots throughout the experiment. A hedonic test was proposed to panellists to judge product acceptability in terms of colour, odour, taste, and aftertaste [32]. This study employed a five-point hedonic scale (1: unacceptable, 2: bad, 3: acceptable, 4: good, 5: excellent).

### 3.8. Statistical Analysis

Each experiment was performed at least in triplicates, and the results were presented as “mean ± standard deviation”. Significant differences between the means of independent variables were examined using the analysis of variance (ANOVA) on IBM SPSS Statistics Software (Version 20.0, IBM SPSS Inc., Armonk, NY, USA) followed by Duncan’s multiple comparison test. Whenever the one-way ANOVA could not be used, the Kruskal–Wallis test was applied after checking for the normal distribution of the groups and the homogeneity of variances. Pearson’s correlation test was conducted to determine the linear correlation among the variables. A value of *p* < 0.05 was considered significant. NemrodW software DesignExpert 8.05b software was used to create and analyse the RSM experiments. Model adequacy was confirmed through ANOVA (checked using the Fisher test), the graphical study of the residuals of the *Y* response, and the r^2^ measurement. Moreover, NemrodW software was used to construct three-dimensional surface plots to investigate the relationship between the response and two independent variables and the desirability study. The visualisation of the sensory analysis data was achieved by Orange Data Mining software (Version 3.92.2, University of Ljubljana Slovene), accessed *via* the Internet at “https://orangedatamining.com/” (accessed on 8 April 2022), as described by [36].

## 4. Conclusions

In this study, the response surface methodology (RSM) employing the Hadamard design was successfully used for optimising the encapsulation of TEO into a stable and homogenous nanoemulsion-based delivery system. The optimum encapsulation conditions were as follows: the percentage of the emulsifier in the continuous phase was 7%, the stirring time was 0 min, the sonication time was 0 min, and the percentage of the dispersed phase was 5%. Under these optimal conditions of encapsulation, the experimental value of the droplet size diameter was 260 nm. The physical characterisation of the optimal nanoemulsion confirmed the stability and the homogeneity of TEO droplets. Considering the encapsulation effect on the antiradical capacity of TEO, results demonstrated a significant amelioration in its capacity to scavenge DPPH^.^ radical after being encapsulated. The antimicrobial efficacy of TEO was improved after its nanoencapsulation since four inhibition zones generated by TNE were statistically larger than those generated by the bulk EO. A milk sensorial analysis highlighted that the general appreciation of the three samples was strongly influenced by the aroma, taste, and aftertaste of milk. The questioned panellists declared that the most appreciated milk was the one enriched with TNE.

## Figures and Tables

**Table 1:** Coded levels and conditions run with the experimental and predicted values used in Response Surface Method experimental design for droplet size diameters (*d*_3,2_) measurements.

Exp	*X*1: Emulsifier Percentage	*X*2: Stirring Time	*X*3: Sonication Time	*X*4: Dispersed Phase Percentage	*d*_3,2_ (nm)	
					Experimental	Predicted
1	−1 (1%)	−1 (0 mn)	−1 (0 mn)	1 (15%)	648.7	660.98
2	1 (7%)	−1 (0 mn)	−0.5 (8 mn)	0.83 (15%)	490.0	470.08
3	1 (7%)	1 (6 mn)	−1 (0 mn)	−1 (5%)	746.4	736.63
4	−1 (1%)	1 (6 mn)	1 (30 mn)	−1 (5%)	1036.0	1046.92
5	1 (7%)	−1 (0 mn)	1 (30 mn)	1 (15%)	463.0	451.88
6	−1 (1%)	1 (6 mn)	−1 (0 mn)	1 (15%)	1012.0	1027.53
7	−1 (1%)	−1 (0 mn)	1 (30 mn)	−1 (5%)	663.5	680.38
8	1 (7%)	1 (6 mn)	1 (30 mn)	1 (15%)	779.5	818.42
9	0 (4%)	0 (3 mn)	0 (15 mn)	0 (10%)	757.6	724.10
10	0 (4%)	0 (3 mn)	0 (15 mn)	0 (10%)	759.0	724.10
11	0 (4%)	0 (3 mn)	0 (15 mn)	0 (10%)	766.9	724.10

1 = maximum level, 0 = medium level and −1 = minimum level.

**Table 2:** Physicochemical characterisation of TNE. Data presented as mean (*n* = 3) ± standard deviation (SD).

TNE	Results	Units
Viscosity	0.10 ± 0.01	mPa/s
Refractive index	1.35 ± 0.08	-
pH	5.10 ± 0.23	-
Droplet diameter	260.8 ± 1.52	nm
PDI	0.26 ± 0.02	-
Turbidity	20.51 ± 0.95	-
Zeta Potential	−10.30 ± 0.78	mV
Conductivity	0.52 ± 0.06	ms/cm

**Table 3:** The antiradical DPPH inhibition capacity (IC, expressed as %) measurement of bulk (TEO) nanoencapsulated (TNE) *T. capitatus* EO (300 µg/mL) and control (C) formed of nanoemulsion components without EO. Values followed by different letters mean statistically significant differences (*p* < 0.01).

	C	TEO	TNE
% IC	<1	40.13 ± 1.58 a	44.28 ± 2.04 b

**Table 4:** Growth inhibition zones (IZ, expressed in mm) were obtained with the bulk (TEO) and nanoencapsulated (TNE) *T. capitatus* EO (33 mg/mL) against five microbial strains. Values followed by different letters mean statistically significant differences (*p* < 0.01).

Microbial Strains		IZ (mm)		Enhancement %
		TEO	TNE	
*Gram+* *Bacteria*	*Staphylococcus aureus*	7.00 ± 0.46 b	10.33 ± 0.27 d	+47.62
	*Enterococcus faecalis*	8.67 ± 0. 6 c	10.33 ± 0.36 d	+19.23
*Gram-* *Bacteria*	*Salmonella typhimurium*	6.00 ± 0.27 a	8.67 ± 0.67 c	+44.44
	*Pseudomonas aeruginosa*	7.67 ± 0.58 cd	8.33 ± 0.77 c	+8.70
*Yeast*	*Candida albicans*	7.67 ± 0.44 cd	12.33 ± 0.46 e	+60.87

**Table 5:** The population distribution of general milk appreciation (expressed as % of the total population). MC: Control milk, MEO: Milk enriched with bulk EO, MNE: Milk enriched with nanoemulsion.

Hedonic Scale		1	2	3	4	5
Population distribution (%)		2.19	23.5	28.96	37.7	7.65
Milk samples	MC	0.55	10.38	10.38	13.31	2.48
	MEO	0	9.29	7.65	9.84	1.84
	MNE	1.64	3.83	10.93	15.15	2.73

**Table 6:** Actual levels at coded factor levels of independent variables used in the RSM.

Symbol	Independent Variables	Actual Levels at Coded Factor Levels		
		−1	0	1
*X*_1_	% emulsifier	1	4	7
*X*_2_	Stirring Time (min)	0	3	6
*X*_3_	Sonication Time (min)	0	15	30
*X*_4_	% dispersed phase	5	10	15

**Table 7:** Hadamard design of different TEO nanoemulsion formulations.

N° Exp	*X*_1_	*X*_2_	*X*_3_	*X*_4_	Emulsifier Concentration	Stirring Time	Sonication Time	Dispersed Phase
					%	min	min	%
1	−1	−1	−1	1	1	0	0	15
2	1	−1	−0.5	0.83	7	0	8	12
3	1	1	−1	−1	7	6	0	5
4	−1	1	1	−1	1	6	30	5
5	1	−1	1	1	7	0	30	15
6	−1	1	−1	1	1	6	0	15
7	−1	−1	1	−1	1	0	30	5
8	1	1	1	1	7	6	30	15
9	0	0	0	0	4	3	15	10
10	0	0	0	0	4	3	15	10
11	0	0	0	0	4	3	15	10

## Data Availability

Not applicable.

## Supplementary Materials

The following supporting information can be downloaded at: https://www.mdpi.com/article/10.3390/molecules27217330/s1, Table S1: Chemical composition of *T. capitatus* essential oil; Table S2: Regression coefficients of the predicted second-order polynomial model for the droplet size measurements; Table S3: Analysis of variance (ANOVA) of the predicted second-order polynomial models for the droplet size measurements.

## Sample Availability

Samples of the compounds are not available from the authors.
