# Supplementary material for: Essential Oil Stabilisation by Response Surface Methodology (RSM): Nanoemulsion Formulation, Physicochemical, Microbiological, and Sensory Investigations
Source: Molecules. 2022 Oct 28;27(21):7330. doi: 10.3390/molecules27217330 (PMC9655826; doi:10.3390/molecules27217330)
Supplement: Supplementary file 1 [file molecules-27-07330-s001.zip › molecules-1938794-supplementary.pdf]

Supplementary material

Table S1. Chemical composition of *T. capitatus* essential oil

Compound	percentage of total volatiles
α-thujene	1.49
α-pinene	0.71
Camphene	0.21
β-myrcene	1.42
I-Phellandrene	0.30
α-terpinene	1.84
p-cymene	7.06
β-phellandrene	0.55
γ-terpinene	7.58
Linalool	1.95
Borneol	0.57
Cyclohexen-1-ol	0.91
Thymol	0.24
Carvacrol	70.62
Caryophyllene	4.55

Table S2. Regression coefficients of the predicted second-order polynomial model for the droplet size measurements.

Terms	Regression coefficients	Standard Error	t Value	Statistical significance
β_0	724.10	1.51	478.94	***
β_1	-129.85	1.77	-73.24	***
B_2	183.27	1.77	103.38	***
B_3	25.30	1.77	14.27	**
B_4	15.60	1.77	8.80	**

*(**;***) is the significance level at p<0.01 ; p<0.001, respectively.*

Table S3. Analysis of variance (ANOVA) of the predicted second-order polynomial models for the droplet size measurements.

Source of variation	SS	DF	MS	*F* value	Statistical significance
**Regression**	4.10674E+0005	4	1.02668E+0005	4083.3248	***
**Residual**	8.11427E+0003	6	1.35238E+0003		
**Validity**	8.06399E+0003	4	2.01600E+0003	80.1802	*
**Error**	5.02867E+0001	2	2.51433E+0001		
**Total**	4.18788E+0005	10			

DF: Degree of freedom; SS: sum of squares; MS: mean square

(*;***) is the significance level at p<0.05 ; p<0.001 respectively.
